# Multiple Bilateral CSF-venous Fistulas in Spontaneous Intracranial Hypotension: A Therapeutic Challenge

**DOI:** 10.1007/s00062-022-01234-2

**Published:** 2022-12-02

**Authors:** Niklas Lützen, Christian Fung, Jürgen Beck, Horst Urbach

**Affiliations:** 1 Dept. of Neuroradiology, Medical Center, University of Freiburg, Breisacher Str. 64, 79106 Freiburg, Germany; 2 Dept. of Neurosurgery, Medical Center, University of Freiburg, Freiburg, Germany

## Introduction

CSF-venous fistulas (CVF) were first described in 2014 and have become increasingly identified as a cause of spontaneous intracranial hypotension (SIH) in up to 25% of patients [1]. The most likely pathomechanism is a rupture of an arachnoid granulation at the level of a nerve root sleeve, leading to a direct connection between the CSF and a paravertebral vein.

In 2021, Schievink et al. [2] reported multiple CVFs in 9/97 (9.3%) and multiple lateral leaks in 4/65 (6.2%) of 745 SIH patients (97 and 65 patients are subgroups of 745 SIH patients), where a co-incidence of different types of leaks was observed in 5 patients.

We report on a severely disabled 56-year-old woman with a frontotemporal brain sagging syndrome who developed 10 CVFs after surgical ligation of a single CVF 6 months before. To our knowledge, this is the first report of more than four fistulas in a single patient. Multiple de novo CVFs represent a therapeutic challenge suggesting that a definite cure of the disease is not possible and low invasive treatment should be adapted to the patient’s symptoms.

## Case Presentation

A now 56-year-old woman had been suffering from orthostatic headaches since 2009. Since 2014 she had not been able to work because symptoms such as frequent vomiting, dysarthria, visual reduction, ataxia, tinnitus and cognitive impairment evolved. Magnetic resonance imaging (MRI) and a CSF opening pressure of 4 cm H_2_0 fulfilled the diagnostic criteria for SIH (International Headache Society 2018) and she was treated with epidural blood patches (EBP) in 2016, 2017 and 2018, and with open surgery at the level of T5 in 2019. A CSF leak had not been found and treatment only temporarily improved clinical symptoms.

**Fig. 1:**
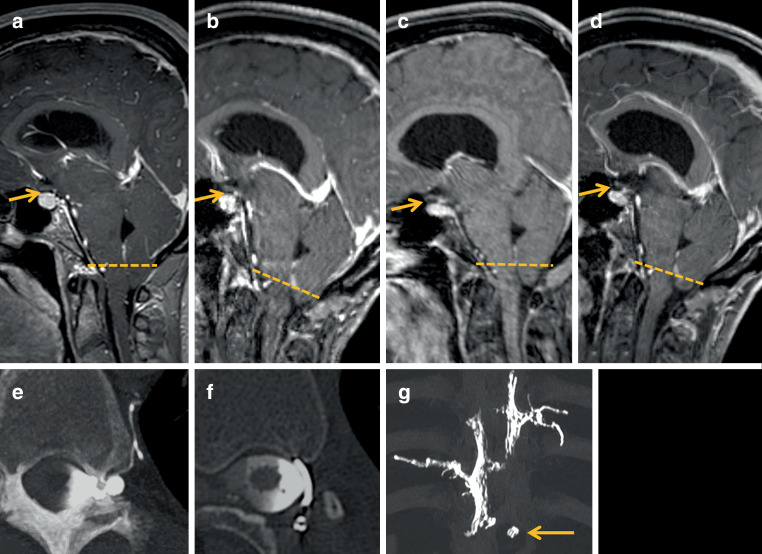
**a** Sagittal MRI from 2017 after 2 epidural blood patches (EBPs). Distinctive MRI signs with a Bern SIH score of 8 (pronounced pachymeninges and engorgement of sinuses are not shown), predominantly with brain sagging, abolished suprasellar distance (*arrow*) and severe tonsillar herniation (*dashed* *line*). **b** MRI in 02/2021 after surgical closure of a CSF-venous fistula (CVF) at T10/11 left with improved tonsillar herniation (*dashed* *line*) while still showing narrow suprasellar distance (*arrow*). **c** MRI 5 months after surgery in 07/2021 improved with Bern SIH score from 8 to 4 (normalization of pachymeninges and sinuses are not shown), widening of suprasellar distance (*arrow*) but worsening of tonsillar herniation (*dashed* *line*). **d** MRI 3 days after transvenous embolization with onyx at T8/9 left and T9/10 right, tonsillar herniation improved again (*dashed* *line*) while mamillopontine distance is only slightly more visible (*arrow*). **e** Paravertebral CVF left at T10/11 on axial LD-CTM with maximum intensity projection (MIP). **f** LD-CTM the same level after surgical ligation (clipping) with hemilaminotomy left shows definitive closure of the CVF. **g** Coronal MIP of a native CT depicts clip material at the level T10/11 left (*arrow*) and embolization onyx cast at the level T8/9 left and T9/10 right

In 08/2020, we encountered an emaciated woman with speech and cognitive impairment as well as facial dyskinesia, who had been bedridden since 2014. The MRI from 2017 (Fig. 1a) showed predominant brain sagging and tonsillar herniation, suggesting the diagnosis of a frontotemporal brain sagging syndrome, for which an underlying cause has rarely been found to date [3]. Myelography and CT myelography did not disclose a CSF leak; she received an additional EBP that worked significantly, but again only temporarily, for a period of 1 week.

In 01/2021, lateral decubitus digital subtraction myelography (LD-DSM) and LD-CTM disclosed a distinct CVF at the level T10/11 left (Fig. 1e). After surgical ligation of the T10/11 CVF, the patient’s symptoms (headache, ataxia, mobility, cognition) and MRI signs significantly improved (Fig. 1b).

**Fig. 2:**
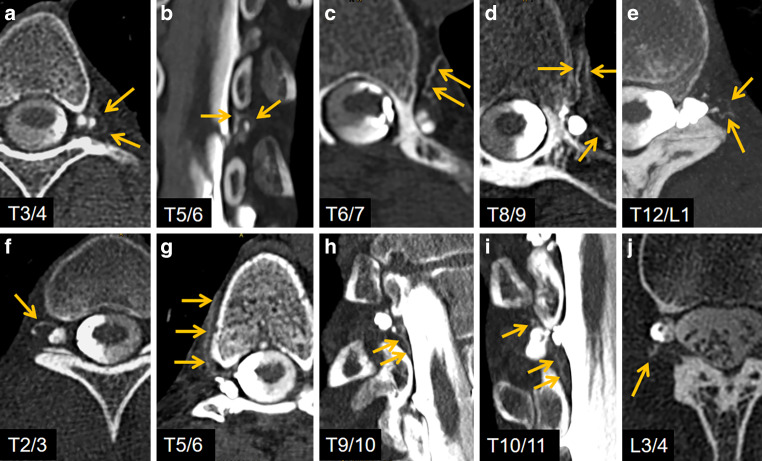
**a–e** Axial and coronal (**b**) LD-CTM of the left side in 07/2021 show 5 different spinal levels with a CVF (respective *orange arrows*). MIP reconstruction at the level T5/6 and T12/L1. **f–j** Axial, oblique coronal (**h**) and coronal (**i**) LD-CTM of the right side in 07/2021 show 5 different spinal levels with a CVF (respective *orange arrows*). MIP reconstruction at the level T5/6, T9/10 and T10/11

Accompanied by orthostatic headache, disturbance, fatigue and a bedriddenness of 4–5 h/day, she came back in 07/2021. Brain sagging on MRI had increased slightly again (Fig. 1c), but overall MRI signs had improved with an SIH score of 4. In LD-CTM, the left-sided T10/11 CVF was proven to be closed (Fig. 1d) but several new de novo CVFs had developed (Fig. 2a–j). We decided to close 2 CVFs by transvenous embolization with Onyx (Fig. 1g). Symptoms significantly improved, especially dysarthria and cognitive impairment. The MRI signs of SIH moderately improved compared to the post-surgery MRI in 02/2021 and significantly to 2017 (Fig. 1d) but 3 months later, in 10/2021, she again complained of worsening of symptoms.

**Fig. 3:**
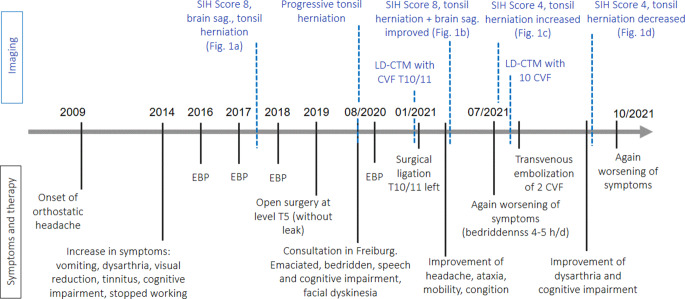
Timeline of patient’s history showing symptoms and therapy compared to imaging

Details of the patient’s medical history are displayed in a graphical timeline (Fig. 3).

## Discussion

CSF-venous fistulas as a cause of SIH can be numerous: there has been a report with four [2], and with two [4] and a few reports with multiple, but not further counted [5], but never a report with more than 10. CVFs can arise de novo at different spinal segments after occlusion of a CVF in another location [2, 6]. Hypotheses for de novo resulting CVFs are underlying connective tissue weakness, idiopathic intracranial hypertension (IIH) with the CVF as “solution” to reduce CSF hypertension [7], and rebound intracranial hypertension (RIH), which occurs in about 25% after closure of a CSF leak [8]. An RIH is usually a transient condition; however, there are severe courses that have been treated with CSF shunting or stenting of the intracranial venous sinus.

Whether a CVF is found at all depends on the accuracy and modality of the diagnostics. In general, DSM is considered to be the best examination technique, although we have found CTM to be very effective; however, most important for diagnostics is the lateral decubitus position, which increases the diagnostic yield fivefold in comparison to the prone position [1]. Some of the CVFs presented in this case are very small but show distinctly contrasted veins (Fig. 2a, e, e.g. at T3/4 and T12/L1). Recent advances in imaging, such as photon counting CT (improved spatial resolution) or dual energy CT (improved iodine contrast) likely increase the detectability of small CVF. Basically, the question remains whether a generally assumed physiological CSF drainage via spinal arachnoid granulations can be distinguished from very small CVFs. At least, such physiological drainage visible on imaging has not yet been described in the literature.

Therapeutic approaches include surgical ligation with a complete long-term resolution in symptoms in approximately 70% [9], transvenous Onyx embolization [4] with an overall clinical outcome of 90% [5], and CT-guided fibrin patches targeting the draining vein with good success rates [9]. In contrast, EBP are barely effective [9].

Even if closure of all CVFs were to be considered, one would face several problems: minimally invasive surgery at multiple segments represents a quite extensive overall procedure. Surgical access from the back of the spine requires a partial or total hemilaminotomy to reach the nerve root sleeve, which may compromise spinal stability. In CT-guided multilevel fibrin patches, it could be difficult to hit the draining veins. Transvenous Onyx embolization is probably the most reasonable therapeutic approach in a scenario like this. As Borg et al. describe [10], the epidural venous plexus is both rich and valveless, once reaching there with a microcatheter, navigation to the point of fistula at multiple levels might be easy and straightforward.

## Conclusion

In a single patient with a frontotemporal brain sagging syndrome, more than 10 CSF-venous fistulas occurred over the course of the disease. Only gradual closure of the fistulas seemed reasonable and was accompanied by temporary improvement of clinical symptoms and on imaging.

## Abbreviations

CSF: Cerebrospinal fluid
CTM: Computed tomography myelography
CVF: CSF-venous fistula
DSM: Digital subtraction myelography
RIH: Rebound intracranial hypertension
SIH: Spontaneous intracranial hypotension
